# The relationship between thiamin, folic acid and cognitive function in a rat model of uremia

**DOI:** 10.1080/0886022X.2024.2329257

**Published:** 2024-03-14

**Authors:** Yifei Lu, Chenqi Xu, Kewei Xie, Bingru Zhao, Minzhou Wang, Cheng Qian, Xuemei Chen, Leyi Gu, Wangshu Wu, Renhua Lu

**Affiliations:** a Department of Pharmacy, Shanghai University of Traditional Chinese Medicine, China; b Department of Nephrology, Renji Hospital, School of Medicine, Shanghai Jiao Tong University, Shanghai, China; c Department of Anesthesiology, Renji Hospital, School of Medicine, Shanghai Jiao Tong University, Shanghai, China

**Keywords:** End-stage renal disease, uremic rat model, cognitive impairment, vitamin B, oxidative stress

## Abstract

End-stage renal disease is a worldwide health burden, but the pathogenesis of uremia-associated cognitive impairment (CI) is poorly recognized. We hypothesized that uremia brings about deficiency of thiamin and folic acid and causes CI by inducing oxidative stress. Therefore, 24 Sprague-Dawley rats were randomly divided into two groups: a 5/6 nephrectomy group (*n* = 12) and a sham-operated group (*n* = 12). The Morris water maze was used to assess the cognitive function eight weeks post-surgery, and serum levels of thiamin, folic acid and homocysteine were detected subsequently. Brain and kidney tissues were collected for pathological examination and 8-Hydroxy-2’-deoxyguanosine (8-OHdG) immunochemistry staining. Results showed that the escape latency on training days 1-2 was longer, and the time in quadrant IV on experimental day 6 was significantly shorter in 5/6 nephrectomy group. Meanwhile, the uremic rats showed decreased thiamin, folic acid and increased homocysteine. We also found the time in quadrant IV was positively correlated with thiamin and folic acid level, while negatively correlated with the blood urea nitrogen and 8-OHdG positive cell proportion. Furthermore, in 5/6 nephrectomy group, the hippocampal neuron count was significantly reduced, and a greater proportion of 8-OHdG positive cells were detected. Pretreating LPS-stimulated rat microglial cells with thiamin or folic acid *in vitro* alleviated the inflammatory impairment in terms of cell viability and oxidative stress. In summary, we applied a uremic rat model and proved that uremia causes serum thiamin and folic acid deficiency, homocysteine elevation, along with neuron reduction and severe oxidative stress in hippocampus, finally leading to CI.

## Introduction

End-stage renal disease (ESRD) is a major health burden worldwide, however, cognitive impairment (CI) is a common but poorly recognized complication in these patients, especially in those on dialysis [1, 2]. Our previous epidemiological study showed that the incidence of CI, as defined as a score <26 by the Montreal Cognitive Assessment test, was as high as 51.6% (113/219) in patients undergoing maintenance hemodialysis (MHD) [3]. The incidence of CI in kidney disease is 2.5 times higher than in non-kidney disease [4]. Due to the complexity of ESRD, the pathogenesis of CI remains unclear. Recent studies have shown that traditional and nontraditional risk factors, as well as dialysis-related risk factors, play important roles in the CI experienced by patients with ESRD. Of these factors, oxidative stress has become a significant research hotspot and is known to cause cardiovascular complications which are one of the major causes of death in ESRD patients [5].

Thiamin and folic acid both belong to the water-soluble vitamin B family. Thiamin, also known as vitamin B1, participates in pentose phosphate pathway as a cofactor for transketolase and plays an important role in stabilizing neuronal membranes as well as reducing reactive oxygen species (ROS) production in the brain [6]. Folic acid, also known as vitamin B9, has antioxidative and endothelial protective properties by directly scavenging free radicals and increasing endothelial nitric oxide synthase (eNOS) coupling to promote NO production [7, 8]. Inhibiting eNOS coupling is exactly the mechanism by which homocysteine induces oxidative stress and endothelial injury. Besides, folic acid is also essential for the conversion of homocysteine to methionine, thus reducing serum homocysteine and the neuronal damage it induced [9]. However, whether thiamin and folic acid are deficient in uremic environment is incompletely understood, and the relationship between these two antioxidants and uremia-associated CI remains unclear.

**Figure 1.**
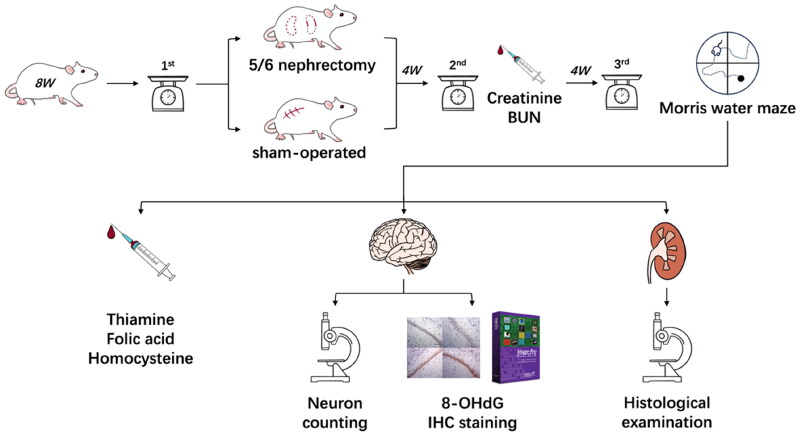
Procedure of the *in vivo* study. BUN, blood urea nitrogen; 8-OHdG, 8-hydroxy-2’-deoxyguanosine; IHC, immunohistochemical.

In this study, we established a rat model of uremia and investigated their cognitive function by Morris water maze. Serum level of thiamin and folic acid in the uremic environment and hippocampal neuron number were also detected. We further determined the degree of oxidative stress in the hippocampus and drew a relationship between these indicators and cognitive function (Figure 1). Moreover, we conducted *in vitro* tests to validate the protective effects of thiamin and folic acid on rat microglial cells independent of uremia.

## Materials and methods

### Induction of uremia in rats

This animal study protocol was reviewed and approved by the animal ethics committee of Renji hospital affiliated to Shanghai Jiao Tong University School of Medicine. 24 Sprague-Dawley (SD) rats were randomly divided into two groups: a 5/6 nephrectomy group (*n* = 12) and a sham-operated group (*n* = 12). At eight weeks old, rats in the former group received 5/6 nephrectomy, as previously reported [10, 11]. Supplementary Figure S1-S4 offered a detailed description of the surgical procedure. Baseline, four-week and eight-week postoperative body weights of rats in both groups were recorded. Serum creatinine and blood urea nitrogen (BUN) were assessed four weeks after the surgery utilizing Hitachi 7600 Series Automatic biochemical analyzer (Hitachi, Japan). Rats were identified as successful uremic models if they met both of the following criteria simultaneously: (1) A total mass of the two resected kidneys should reach 0.74% of their body weight. (2) Four weeks after 5/6 nephrectomy, serum creatinine and BUN levels should be 2-3 times higher than those of sham-operated group [10].

### Assessment of cognitive function in rats

Cognitive function of rats was assessed by Morris water maze, eight weeks after two stage operation. Morris water maze is a circular pool with diameter of 180 cm and height of 40 cm (Supplementary Figure S5) [12]. Water temperature, indoor light and reference objects remained optimal and unchanged. The circular pool was divided into four quadrants, and a transparent glass escape platform of 11 cm in height was placed in the center of quadrant IV. Black ink was poured into the pool and the water level was kept 2 cm higher than the platform to hide it. The animal behavior analysis system equipped with automatic camera (Shanghai Jiliang software, China) was used to track and time the swimming process of rats. Definitions of Morris water maze terms are as follows: (1) Escape latency refers to the time required for rats to find the escape platform in quadrant IV during the first 5 days of training. (2) Time in quadrant IV refers to the total time staying in quadrant IV after starting from NW point on day 6 of experiment. (3) Percentage of time in quadrant IV refers to the percentage of time in quadrant IV to the entire experiment time (90s) on day 6 of experiment. Time in quadrant IV and percentage of time in quadrant IV were used to evaluate the spatial learning and memory function of rats.

The Morris water maze experiment lasted for 6 days. Firstly, each rat was trained for 5 days continuously, four times a day starting from different position set randomly in advance (Supplementary Table S1). When the training began, the rat was put into the pool and supposed to search for the escape platform in quadrant IV within 90s and stay on the platform for 10s. The escape latency was recorded. If the rat failed to find the platform within 90s, it would be gently guided to the platform and asked to stay for 30s. The escape latency under this circumstance would be recorded as 90s. On day 6 of experiment, the escape platform in quadrant IV was removed, and the rat was set to start from NW point, the furthest point from the original escape platform. The time in quadrant IV and percentage of time in quadrant IV were recorded.

### Detection of serum thiamin, folic acid and homocysteine

After completing the Morris Water maze experiment, all rats were euthanized and blood was collected from right atrium to detect serum levels of thiamin, folic acid and homocysteine. Serum thiamin and folic acid were detected on LK3000VI vitamin detector (Tianjin Lanbiao, China) and UniCel DxI 800 Access Immunoassay System (Beckman Coulter, USA) respectively. Homocysteine level was detected on Hitachi 7600-020 automatic biochemical analyzer (Hitachi, Japan).

### Histological examination of the brains and kidneys

Brain and kidney specimens were obtained from randomly selected euthanized rats in both groups. Brain specimen sections of 3 μm thickness were Nissl stained and the number of neurons in the hippocampus was counted manually to determine the degree of brain damage in uremic rats. Anti-8-hydroxy-2′-deoxyguanosine (8-OHdG) (Abcam, US) and HRP-labeled goat anti-mouse IgG (Beyotime, China) were utilized sequentially for immunohistochemical (IHC) staining on hippocampus sections of 2 μm thickness. 8-OHdG positive staining was defined as a brown signal in cytoplasm [13]. Entire hippocampal sections [including CA1, CA2, CA3, CA4 and dentate gyrus (DG) region) were observed under 400× magnification. The number of total cells and 8-OHdG positive cells in the hippocampus were counted and the proportion of positive cells was calculated. To further evaluate oxidative stress in hippocampus semi-quantitatively, Image-Pro Plus analysis was performed by a blinded technician. The specimen sections were shot at 100× magnification and imported into the software. Integrated optical density (IOD) value was output for inter-group comparison [14]. Kidney specimens were stained by periodic acid Schiff (PAS) reagent and observed under optical microscope to determined pathological injury in uremic model rats.

### Cell culture and treatments

Rat microglial cells (Owoto Biotech, China) were cultured in Dulbecco’s Modified Eagle medium (DMEM; KeyGEN Biotech, China) supplemented with 10% FBS (Gibco, US). The cells were pretreated with either benfotiamine (50 or 100 μmol/L; MCE, US) for 24 h, or folic acid (5 or 10 ug/mL; MCE, US) for 1 h, and then stimulated with 1 μg/mL LPS (MCE, US) for 24 h.

### Cell viability assessment

Cell viability was determined by CCK-8 cell proliferation detection kit (KeyGEN Biotech, China) according to the manufacturer’s protocol. In brief, cells (1 × 10^4^ cells/mL) were plated onto 96-well plates, and then subjected to pretreatments and LPS stimulation as described above. Then, the culture media were carefully removed and 10 μL of CCK-8 in 100 μL of medium was added to each well. The cells were incubated for 2 h. In the control group, untreated and unstimulated cells were cultured and incubated under the same conditions. The cell viability was calculated according to the following formula: cell viability (%) = [OD_450_ (treatment)-OD_450_ (blank)]/[OD_450_ (control)-OD_450_ (blank)] × 100.

### ELISA

To detect the concentration of 8-OHdG in the cell culture supernatants, 8-OHdG ELISA kit (Elabscience, China) was used according to the manufacturer’s protocol.

### Statistical analysis

SPSS 20.0 was used for statistical analysis. Data of normal distribution were expressed as mean ± standard deviation. Non-normal data were expressed as medians and interquartile range. T-test, one-way ANOVA and Kruskal–Wallis H test were used for inter-group comparison according to the number of groups and data distribution. Bonferroni’s correction was used for post-hoc test. Pearson’s correlation analysis was used to identify the factors associated with time in quadrant IV of the Morris Water maze. The level of statistical significance was set at *p* < 0.05.

## Results

### Successful model establishment of uremic rats

In the present study, the average body weight of rats in 5/6 nephrectomy group was 201.51 ± 13.37g. The average weight of resected 2/3 left kidney and entire right kidney was 0.48 ± 0.14g and 1.61 ± 0.33g respectively, accounting for 1.04% of the body weight in total. Meanwhile, the serum creatinine and BUN in 5/6 nephrectomy group were significantly higher than those in sham-operated group 4 weeks after operation (104.25 ± 28.85 μmol/L *vs* 37.50 ± 2.12 μmol/L, *p* < 0.001 for serum creatinine; 25.68 ± 3.90 mmol/L *vs* 8.00 ± 0.00 mmol/L, *p* < 0.001 for BUN). Sufficient tissue resection and significant renal function impairment proved that the uremic rat models were successfully established. Detailed body weight data and the results of kidney histological examination were shown in Supplementary Table S2 and Figure S6.

### Comparison of Morris water maze experiment results between the two groups

**Figure 2.**
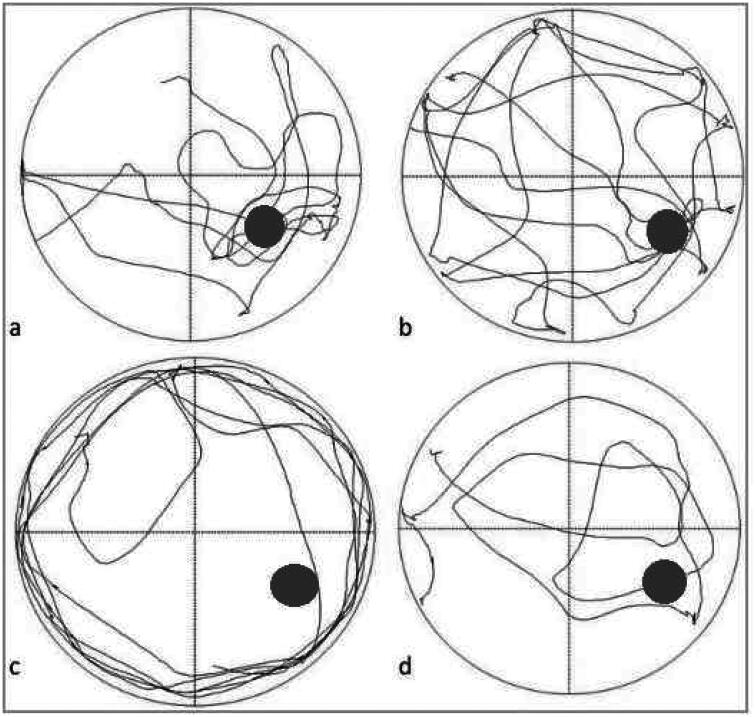
Track map of Morris water maze on day 6. a and b, the track map of Morris water maze in sham-operated group; c and d, the track map of Morris water maze in 5/6 nephrectomy group.

**Figure 3.**
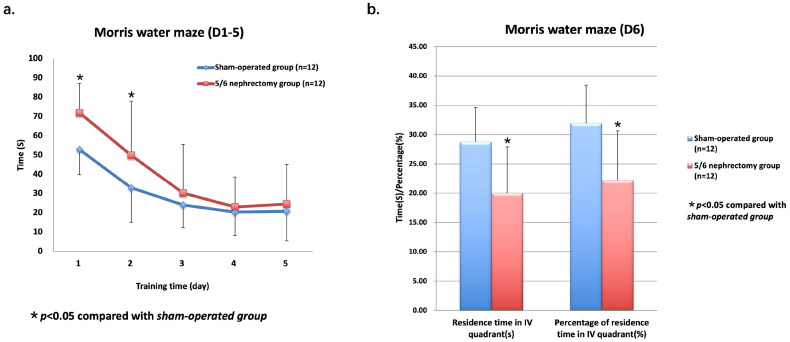
Escape latency of Morris water maze in sham-operated group and 5/6 nephrectomy group. a, training day 1-5. Specific data were shown in Supplementary Table S3. *: *p* < 0.05. b, experimental day 6. *: *p* < 0.05.

The tracks of the rats swimming to find the escape platform in Morris water maze were recorded and timed (Figure 2). The results showed that the escape latency of 5/6 nephrectomy group on training day 1 and day 2 was significantly higher than that of sham-operated group (71.89 ± 15.21s *vs* 52.86 ± 13.05s, *p* = 0.030 for Day 1; 49.86 ± 28.03s *vs* 33.02 ± 17.98s, *p* = 0.046 for Day 2; Figure 3(a)). The escape latency on training day 3-5 was still higher in 5/6 nephrectomy group; however, the differences were nonsignificant (Figure 3(a), Supplementary Table S3). In addition, we found that as training progressed, the escape latency gradually shortened in both groups.

On day 6 of Morris water maze experiment, the time in quadrant IV decreased significantly in 5/6 nephrectomy group compared with sham-operated group (20.00 ± 7.91s *vs* 28.75 ± 5.85s, *p* = 0.005), as well as and percentage of time in quadrant IV (22.23 ± 8.78% *vs* 31.95 ± 6.50%, *p* = 0.006; Figure 3(b)).

### Comparison of thiamin, folic acid and homocysteine level between the two groups

Serum thiamin and folic acid level in 5/6 nephrectomy group were significantly lower than those in sham-operated group (59.49 ± 4.09 nmol/L *vs* 66.03 ± 5.63 nmol/L, *p* = 0.008 for thiamin; 97.53 ± 32.36 ng/mL *vs* 169.29 ± 26.37 ng/mL, *p* < 0.001 for folic acid). Additionally, serum homocysteine in 5/6 nephrectomy group increased compared with sham-operated group (12.99 ± 1.28 μmol/L *vs* 11.63 ± 1.43 μmol/L, *p* = 0.044).

### Comparison of hippocampal neuron count between the two groups

Number of neurons in hippocampal CA1, CA2, CA3, CA4 and DG region in 5/6 nephrectomy group was significantly lower than that in sham-operated group (Table 1). Results of hippocampus histological examination were shown in Supplementary Figure S7.

**Table 1.** Hippocampal neuron count in two groups.

	5/6 nephrectomy group (*n* = 12)	sham-operated group (*n* = 12)	*p* value
CA1	135.00 ± 10.58	189.00 ± 23.00	0.004
CA2	169.00 ± 16.37	199.60 ± 14.47	0.032
CA3	149.33 ± 10.12	182.00 ± 21.39	0.027
CA4	269.67 ± 114.03	562.80 ± 79.23	0.005
DG	428.00 ± 86.54	642.00 ± 95.20	0.019

### Comparison of 8-OHdG IHC staining in hippocampus between the two groups

**Figure 4.**
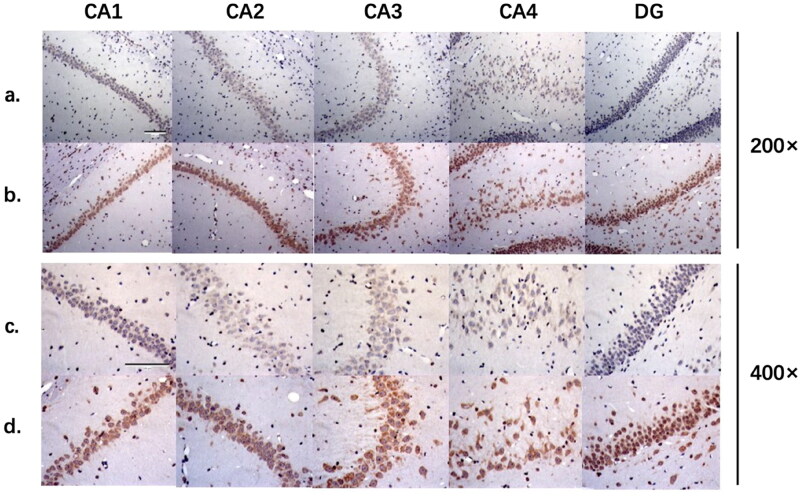
8-OHdG immunohistochemistry staining of hippocampus in two groups. a, sham-operated group (200×); b, 5/6 nephrectomy group (200×); c, sham-operated group (400×); d, 5/6 nephrectomy group (400×). CA1-CA4, the CA1-CA4 region of hippocampal gyrus; DG, dentate gyrus.

8-OHdG positive neurons were observed generally in hippocampal CA1, CA2, CA3, CA4 and DG regions of rats from 5/6 nephrectomy group, while no 8-OHdG positive neuron was seen in sham-operated group (Figure 4). We also analyzed the IHC staining results semi-quantitatively. The proportion of 8-OHdG positive cells in hippocampal CA1, CA2, CA3, CA4 and DG regions from 5/6 nephrectomy group was significantly higher than those from sham-operated group, as well as IOD value assessed by Image-pro Plus (Table 2). These results implicated the presence of oxidative stress in the brain of uremic rats, promoting the pathogenesis of uremia-associated CI.

**Table 2.** Cell counts and IOD values of 8-OHdG IHC staining in two groups.

	sham-operated group				5/6 nephrectomy group			
	Cell count	Positive cell count	Positive cell proportion (%)	IOD value	Cell count	Positive cell count	Positive cell proportion (%)	IOD value
CA1	357	110	30.81	12555	268	146	54.48*	16438*
CA2	344	86	25.00	9486	329	220	66.87*	27611*
CA3	324	78	24.07	9093	249	216	86.75*	27771*
CA4	361	89	24.65	10624	356	272	76.40*	20230*
DG	271	101	37.27	16192	412	219	53.16*	19003*

*: *p* < 0.05 compared with sham-operated group.

### Correlation analysis on time in quadrant IV of Morris water maze

**Figure 5.**
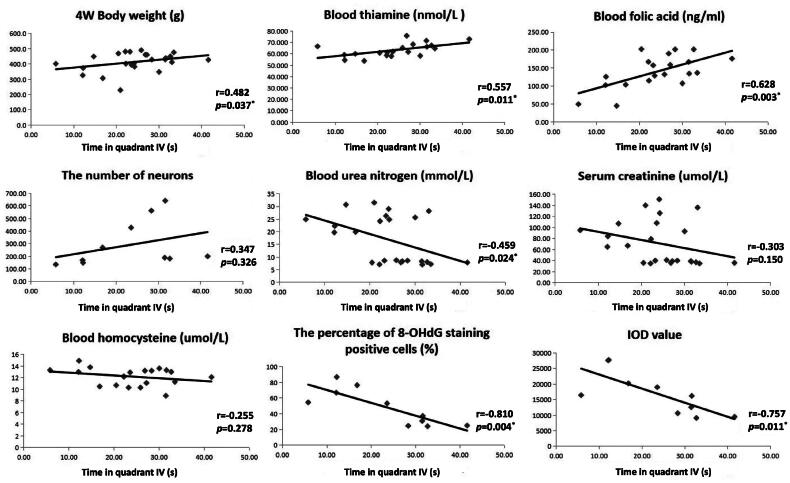
Correlation analysis on time in quadrant IV. r, correlation coefficient; *p*, significance of correlation analysis. *: *p* < 0.05.

Correlation analysis was conducted to identify factors associated with cognitive function of uremic rats (Figure 5). It turned out that four-week postoperative body weight, thiamin and folic acid were positively associated with time in quadrant IV, whereas BUN, 8-OHdG positive cell proportion and IOD value in 8-OHdG IHC staining were negatively associated with time in quadrant IV [*r* = 0.482, *p* = 0.037 for four-week postoperative body weight; *r* = 0.557, *p* = 0.011 for thiamin; *r* = 0.628, *p* = 0.003 for folic acid; r=-0.459, *p* = 0.024 for BUN; r=-0.810, *p* = 0.004 for 8-OHdG positive cell proportion; r=-0.757, *p* = 0.011 for IHC IOD value].

### Protective effects of thiamin and folic acid on rat microglial cells in vitro

**Figure 6.**
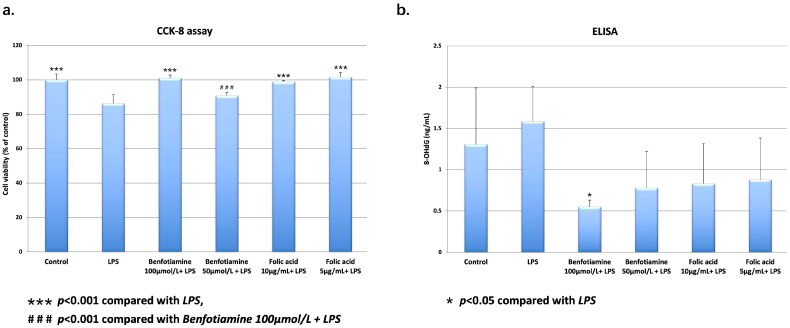
Cell viability and oxidative stress level of LPS-stimulated microglial cells. a, relative cell viability of LPS-stimulated microglial cells pretreated with either benfotiamine or folic acid. ***: *p* < 0.001. b, 8-OHdG concentration in culture supernatants of LPS-stimulated microglial cells pretreated with either benfotiamine or folic acid. Specific data were shown in Supplementary Table S4. *: *p* < 0.05.

To validate the relationship between thiamin, folic acid and cognitive impairment, we tested the effect of benfotiamine and folic acid on LPS-stimulated rat microglial cells *in vitro*. The results of CCK-8 assay showed that LPS stimulation significantly reduced cell viability (86.37 ± 5.14% *vs* 100.00 ± 3.45%, *p* < 0.001; Figure 6(a)). Benfotiamine pretreatment showed a concentration-dependent protective effects on LPS-stimulated microglial cells (101.11 ± 1.71% *vs* 90.82 ± 1.79%, *p* < 0.001; Figure 6(a)). Pretreatment with both high and low concentrations of folic acid preserved cell viability, similar to that of the control group. There was no significant difference observed between the two folic acid groups (98.94 ± 0.75% *vs* 101.67 ± 2.72%, *p* > 0.05; Figure 6(a)).

Additionally, we tested the concentration of 8-OHdG in the culture supernatants of six groups. The 8-OHdG level of the LPS group was slightly higher than that of the control group, but the difference was nonsignificant (1.59 ± 0.42 ng/mL *vs* 1.30 ± 0.69 ng/mL, *p* > 0.05; Figure 6(b)). Pretreatment with either benfotiamine or folic acid tended to alleviate the LPS-induced oxidative stress induced, and high concentration of benfotiamine reached statistical significance (0.55 ± 0.08 ng/mL *vs* 1.59 ± 0.42 ng/mL, *p* = 0.014; Figure 6(b)). Detailed results of ELISA were presented in Supplementary Table S4.

## Discussion

In the present study, we established a rat model of uremia by 5/6 nephrectomy and assessed cognitive function by Morris water maze experiment. Compared with sham-operated group, the escape latency during training was significantly prolonged, while time in quadrant IV on experimental day was significantly reduced in 5/6 nephrectomy group, which indicated that the spatial learning and memory capacity was impaired in uremic rats. Furthermore, the serum levels of thiamin and folic acid in 5/6 nephrectomy group were significantly lower than those in sham-operated group, while homocysteine exhibited an opposite variation trend. Histological examination and IHC staining further revealed that the number of neurons in hippocampus was significantly reduced in 5/6 nephrectomy group, together with increased local oxidative stress indicated by accumulation of 8-OHdG positive neurons. Correlation analysis suggested cognitive function in uremic rats was positively correlated with body weight, blood thiamin and folic acid, while negatively correlated with BUN and the degree of hippocampal oxidative stress. The protective effects of thiamin and folic acid on cognitive function were further confirmed on LPS-stimulated microglial cells.

Although various experiments have been utilized on cognitive function assessment, Morris water maze, designed to evaluate the hippocampal-dependent spatial learning and memory ability of rodents, is the most classical and acknowledged one [15, 16]. Hence, we applied Morris water maze in the current study and revealed significant CI in uremic rats. Our findings are consistent with previous studies. Fujisaki K established uremic models on C57BL/6 mice by left kidney resection and 2/3 right kidney electrocoagulation, and evaluated the cognitive function by radial arm water maze. The researchers observed working memory function impairment after eight weeks of modeling in 5/6 nephrectomy group [17]. Therefore, we hypothesized that the uremic milieu could cause CI in our model rats.

Oxidative stress is considered a major cause of neurodegenerative diseases including cognitive impairment [5]. Previous study reported oxidative stress in the brain tissue of uremic mice, especially in hippocampus. After intervention with antioxidant tempol, the oxidative stress in brain was alleviated and the cognitive function of uremic mice was improved [17]. Homocysteine is acknowledged as an important agent of oxidative stress *in vivo*, because it is susceptible to auto-oxidation with the secondary generation of ROS, and has inhibitory ability on antioxidant enzymes such as glutathione peroxidase (GPx) and superoxide dismutase (SOD) [18]. In our study, blood homocysteine level in 5/6 nephrectomy group was significantly higher than that in sham-operated group, and histological examination revealed a considerable reduction in hippocampal neurons together with the presence of oxidative stress indicated by 8-OHdG IHC staining. Thus, we supposed oxidative stress to play an important role in mechanism of uremia-associated CI.

Thiamin and folic acid are crucial antioxidants in body, and the latter is an essential material for homocysteine metabolism [9]. The intracellular concentration of superoxide and hydrogen peroxide increased significantly, and both the expression and activity of GPx were inhibited in thiamin deficient rats, which can lead to oxidative stress in the brain and various neurological diseases [19]. Clinical studies have reported that the folic acid and vitamin B supplementation can reduce blood homocysteine level and improve cognitive performance in patients with Alzheimer’s diseases or mild cognitive impairment, although this cognitive protective effect has not been demonstrated in ESRD by randomized controlled trials yet [8, 20–23]. In aggregate, these findings proved that lack of thiamin and folic acid is one of the main causes of oxidative stress. Serum level of thiamin and folic acid in 5/6 nephrectomy group was significantly lower than that in sham-operated group in our study, which was consistent with previously reported folic acid and vitamin B deficiency in ESRD patients, as a comprehensive results of metabolic alterations, comorbidities, reduced dietary intake as well as aberrant gastrointestinal motility, mucosal permeability and microbiota [24]. Previous studies demonstrated that the cellular uptake of folic acid is depressed in uremic milieu due to anionic inhibition, thus serum folic acid does not reflect the body storage or intracellular use, but mirrors the recent dietary intake level [8, 24]. In our study, rats in the two groups were given the same diet, while the body weight of 5/6 nephrectomy group increased much more slowly than that of sham-operated group, and the serum folic acid decreased significantly. These results suggested that the uremic model rats suffered from dietary deficiency of folic acid. Given that most molecular mediators associated with brain damage are retained in the circulation and act through it, reduced serum folic acid could partially explain the increased oxidative stress in brain and CI in uremic rats [1]. More importantly, linear correlation was observed between blood thiamin, folic acid and time in quadrant IV of Morris water maze, further implicating the protective effect of thiamin and folic acid on cognitive function in uremic rats. In addition to animal models, pretreatment of microglial cells with either of these two antioxidants could reduce LPS-induced inflammatory impairment, as indicated by preserved cell viability and reduced 8-OHdG production, validating their effects independent of uremia.

This study has some limitations. Firstly, we found the statistical association between thiamin, folic acid and cognitive function and confirmed it by *in vitro* tests, however more research are required to reveal the underlying mechanisms. Secondly, we did not use nutrient supplemented uremic models to draw a more robust causal relationship. Thirdly, we did not apply dialysis on uremic model rats so whether dialysis plays a role in uremia-associated CI remains unclear.

In summary, we proved that uremia leads to thiamin and folic acid deficiency, as well as increased homocysteine and oxidative stress in hippocampus, resulting in neuron contraction and CI. Serum thiamin and folic acid, positively correlated with cognitive function, are the potential therapeutic targets for CI in ESRD patients.

## Supplementary Material

Supplemental Material
