# Supplementary material for: The relationship between thiamin, folic acid and cognitive function in a rat model of uremia
Source: Ren Fail. 2024 Mar 14;46(1):2329257. doi: 10.1080/0886022X.2024.2329257 (PMC10946272; doi:10.1080/0886022X.2024.2329257)
Supplement: Supplemental Material [file IRNF_A_2329257_SM6146.pdf]

Supplementary

Production of uremic rat model

All rats received surgery under abdominal anesthesia with 2% pentobarbital sodium (0.3ml/100g). For rats in 5/6 nephrectomized group, the left kidney was exposed on sterilized towel, and 2/3 of it was resected carefully to avoid bleeding (Figure S1-S2). One week after the first stage operation, we performed the second stage operation to remove the right kidney (Figure S3). The excised normal kidney tissue was preserved in 4% paraformaldehyde (Figure S4). Rats in sham-operated group received a sham operation including decapsulation of both kidneys but no tissue was resected. After each surgical procedure, the incisions were closed in layers with clips applied to the skin. Meanwhile, 100,000 Unit penicillin sodium and 8,000 Unit gentamicin were injected into thigh muscle of each rat for 3 days continuously to prevent infection. All surgical procedures were performed by the same investigator.

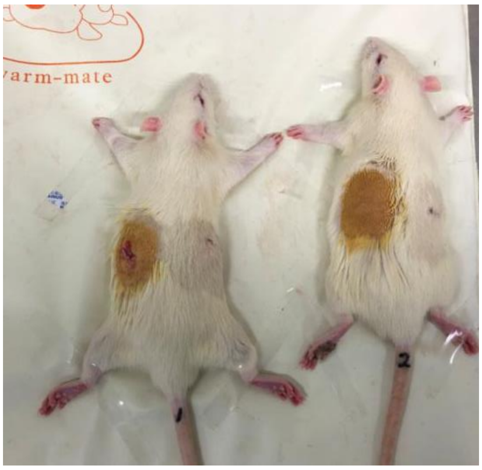

Figure S1: Skin disinfection

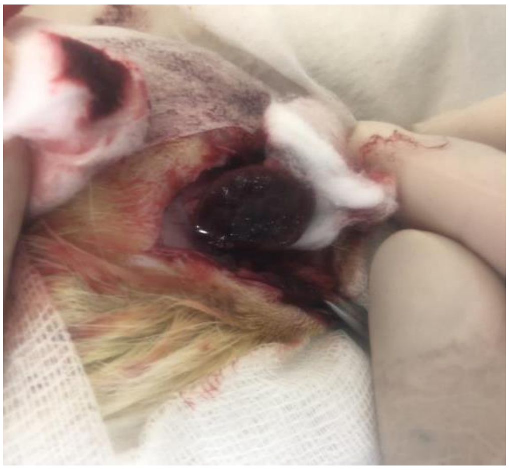

Figure S2: Resect 2/3 left kidney tissue

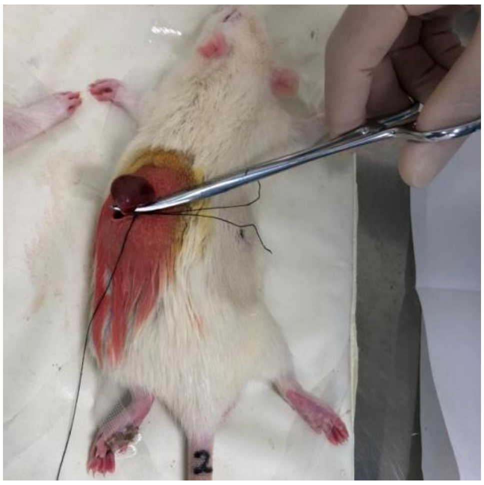

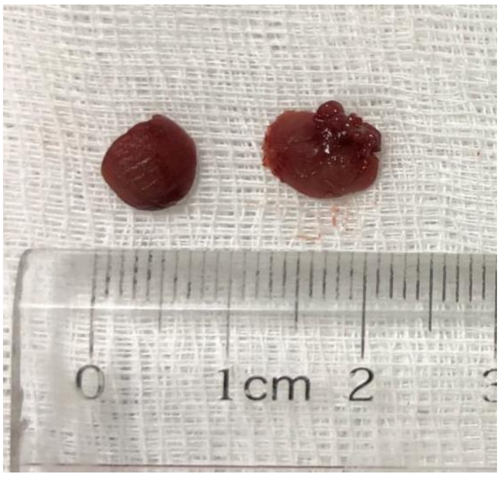

Figure S3: Resect right kidney **Figure S4: Resected kidney tissue**

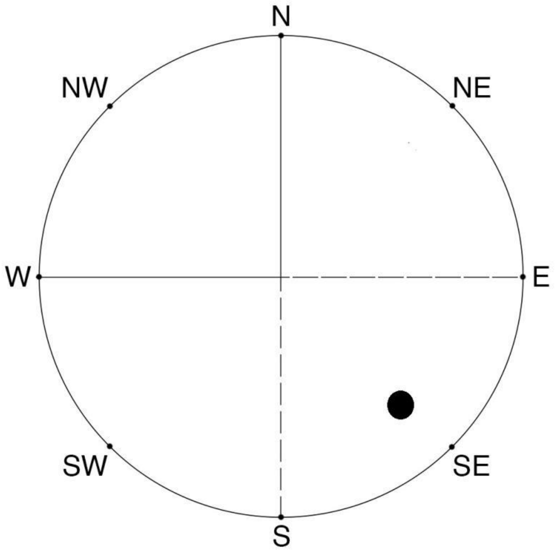

Figure S5: Plan of Morris water maze

Table S1: Starting position of Morris water maze[*].

Day	Trial 1	Trial 2	Trial 3	Trial 4
1	SW	NE	W	N
2	W	SW	N	NE
3	N	W	NE	SW
4	NE	N	SW	W
5	SW	W	NE	N
6 (Probe)	NW			

*: As S, E, SE points were too close to the escape platform in quadrant IV, these three points were excluded from the alternatives of random starting position.

Table S2: Body weight of rats in two groups.

	5/6 nephrectomy group (n=12)	sham-operated group (n=12)	*p* value (inter-group)
Baseline (g)	201.51±13.37	213.40±5.37	0.097
4-week post-operation (g)	384.75±37.50 [0.032] [*]	428.00±1.41 [0.002]	0.002
8-week post-operation (g)	444.25±27.67 [0.035]	525.50±33.23 [0.025]	0.025

*: *p* value compared with body weight at last measure point is shown in square brackets.

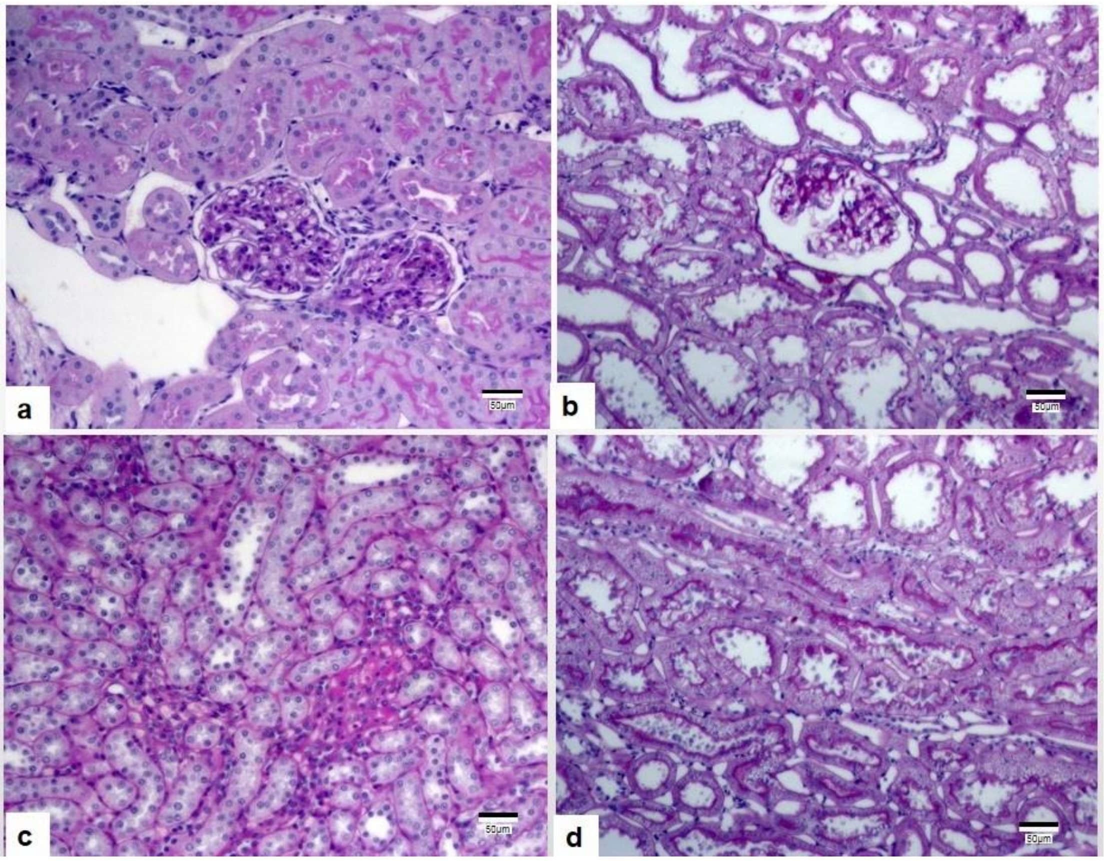

Figure S6: PAS staining of kidney tissue in rats from two groups. a, glomeruli of sham-operated group. b, glomerulus of 5/6 nephrectomy group; the glomerular mesangial cells and matrix segments were slightly proliferated. A capillary loop was adhered to Bowman's capsule and presented segmental sclerosis. c, renal tubules of sham-operated group. d, renal tubules of 5/6 nephrectomy group; severe tubule interstitial lesions was observed, manifested by multifocal atrophy and compensatory dilatation of tubules, as well as interstitial inflammatory cell infiltration.

Table S3: Escape latency of Morris water maze training (D1-D5) in two groups.

	5/6 nephrectomy group (n=12)	sham-operated group (n=12)	*p* value
Day1 (s)	71.89±15.21	52.86±13.05	0.030
Day2 (s)	49.86±28.03	33.02±17.98	0.046
Day3 (s)	30.27±25.30	24.04±11.78	0.451

Day4 (s)	23.04±15.63	20.38±12.18	0.647
Day5 (s)	24.47±20.70	20.78±15.29	0.624

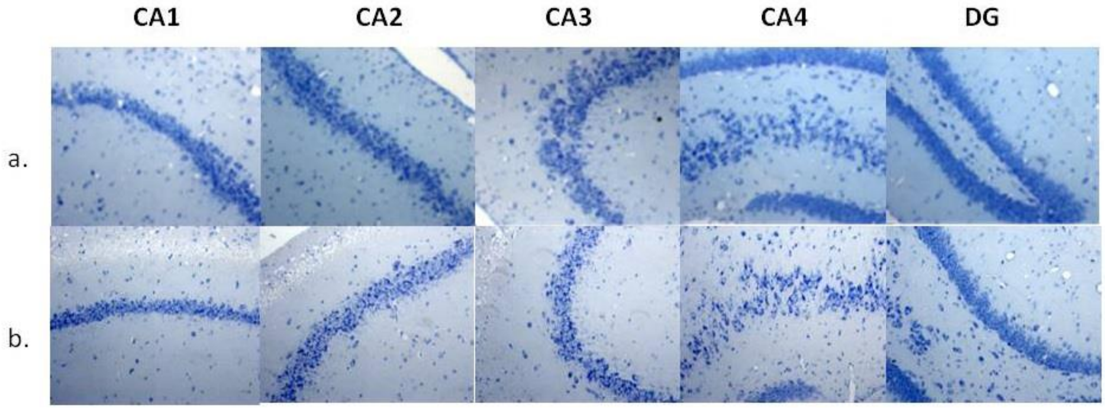

Figure S7: **Nissl staining of hippocampus tissue in rats from two groups**. a, sham-operated group; b, 5/6 nephrectomy group. CA1-CA4, the CA1-CA4 region of hippocampal gyrus; DG, dentate gyrus.

Table S4: 8-OHdG concentration in the culture supernatants of six groups.

	8-OHdG concentration (ng/mL)	*p* value* (compare with LPS group)
Control	1.30±0.69	>0.999
LPS	1.59±0.42	—
Benfotiamine 100µmol/L+ LPS	0.55±0.08	0.014
Benfotiamine 50µmol/L+ LPS	0.78±0.44	0.181
Folic acid 10µg/mL+ LPS	0.83±0.49	0.501
Folic acid 5µg/mL+ LPS	0.88±0.51	0.501

* *p* value is adjusted by Bonferroni's correction for multiple tests.
